# Diagnostic Delay in Amyotrophic Lateral Sclerosis: A Systematic Review and Meta-Analysis Comparing Time From Symptom Onset to Diagnosis in Bulbar-Onset Versus Limb-Onset Disease

**DOI:** 10.7759/cureus.113188

**Published:** 2026-07-22

**Authors:** Yousaf Saeed, Maryam Fatima, Munib ur Rehman, Muhammad Hamza Aslam

**Affiliations:** 1 Internal Medicine, Tehsil Headquarter Hospital, Liaquatpur, PAK; 2 Internal Medicine, United Health Services Hospital, Johnson City, USA; 3 Pediatric Neurosurgery, Birmingham Women’s and Children’s NHS Foundation Trust, Birmingham, GBR; 4 Internal Medicine, Shaikh Khalifa Bin Zayed Al Nahyan Medical and Dental College, Lahore, PAK

**Keywords:** amyotrophic lateral sclerosis, bulbar onset, diagnostic delay, limb onset, meta-analysis

## Abstract

Diagnostic delay is a recognized challenge in amyotrophic lateral sclerosis (ALS), depriving patients of timely access to disease-modifying therapy and multidisciplinary care. Although several individual cohorts have reported diagnostic delay separately for bulbar-onset and limb-onset ALS, few studies have directly compared the two, and their estimates have not been pooled. We aimed to compare time from symptom onset to diagnosis in bulbar-onset versus limb-onset ALS. We searched PubMed and Cochrane CENTRAL (Cochrane Central Register of Controlled Trials) from inception through April 2026 for studies reporting diagnostic delay separately for the two onset types. Where upper- and lower-limb onset were reported separately, these were combined into a single limb-onset group using standard formulae; medians with interquartile ranges were converted to means and standard deviations using the method of Wan et al. A random-effects meta-analysis (DerSimonian-Laird) pooled studies reporting a usable measure of dispersion by onset group, with the remaining studies summarized narratively. The outcome was the mean difference (MD) in diagnostic delay in months, where a negative value indicates faster diagnosis in bulbar onset; heterogeneity was quantified with I² and risk of bias with the Newcastle-Ottawa Scale. In total, 13 studies (898 bulbar-onset and 2,438 limb-onset patients from eight countries) met the inclusion criteria; nine contributed to the meta-analysis, as four reported no usable measure of dispersion and were summarized narratively. Bulbar-onset patients were diagnosed significantly faster than limb-onset patients (MD = −4.42 months; 95% confidence interval −5.73 to −3.11; p < 0.001), with moderate heterogeneity (I² = 56%). The direction of effect was consistent across all studies, and the pooled estimate was stable on leave-one-out analysis. The four non-pooled studies were each directionally consistent. Bulbar-onset ALS is diagnosed approximately 4 months faster than limb-onset ALS, likely because distinctive bulbar symptoms prompt earlier specialist referral whereas limb weakness is attributed to more common musculoskeletal or orthopedic conditions. Strategies raising awareness of limb-onset ALS among primary care and orthopedic physicians are warranted.

## Introduction and background

Amyotrophic lateral sclerosis (ALS) is a neurodegenerative disease in which the upper and lower motor neurons slowly die, usually following a fast and fatal course. Worldwide incidence is close to 2 cases per 100,000 people per year [1,2], and the estimated lifetime risk is about 1 in 350 [3]. Because most patients survive only two to four years after symptoms begin [1,3], the time spent reaching a diagnosis uses up part of the limited period in which treatment and planning are still possible.

Amyotrophic lateral sclerosis is divided into forms by where weakness first appears. Bulbar-onset ALS (about 25% to 30% of cases) begins with slurred speech, trouble swallowing, or changes in the voice [1]. Limb-onset ALS (about 60% to 70%) begins with weakness in an arm or a leg [1]. A smaller group first develops breathing problems. Onset site also carries prognostic weight, and bulbar-onset patients have generally been reported to have shorter survival from symptom onset [3,4].

Diagnosing ALS is inherently slow. There is no confirmatory test; the diagnosis is made clinically using established criteria, El Escorial, Revised El Escorial, Awaji, and, more recently, Gold Coast, which require signs of both upper and lower motor neuron damage and, in most versions, evidence that the disease is spreading [1,3]. Because this often depends on watching the disease evolve and ruling out look-alike conditions, it frequently cannot be made at a single visit.

As a result, diagnostic delay is a well-recognized problem. Patients commonly see several specialists before the diagnosis is confirmed, and the average time from first symptom to diagnosis has been reported at roughly 9 to 18 months across different health systems [3]. This interval has more than one stage: the time a patient takes to seek help, and the often longer time the health system takes to refer the patient and reach the diagnosis. Where the delay builds up matters, because different stages call for different solutions.

Much of the delay comes from mistaking early ALS for commoner conditions. Bulbar-onset ALS is often mistaken for stroke, a functional neurological disorder, myasthenia gravis, or ear, nose, and throat (ENT) disease [3]. Limb-onset ALS is frequently blamed on cervical or lumbar spine disease, peripheral neuropathy, or orthopedic problems, so these patients are often routed through orthopedic, spine, and rehabilitation services before reaching a neurologist [1]. Because the two forms present so differently, and are mistaken for different things, there is good reason to expect they are not diagnosed at the same speed.

A delayed diagnosis carries real costs. Disease-modifying drugs are started late - riluzole first and most widely, with edaravone and, for superoxide dismutase 1 (SOD1)-related disease, tofersen also available [3,5] - multidisciplinary care is delayed, less time remains for advance care planning, and patients and families carry a heavy emotional burden during the uncertainty. In a disease measured in months, avoidable delay directly shrinks the window for treatment, support, and planning.

Several studies have reported diagnostic delay separately for bulbar-onset and limb-onset ALS, but this evidence has never been pooled, and the figures vary widely between settings [3], leaving clinicians without a single combined estimate of how much the two forms differ. This matters for two reasons. First, a single pooled estimate gives clinicians a concrete figure to set expectations and counsel patients, rather than the widely varying numbers from individual cohorts. Second, and more importantly, if one form is consistently diagnosed later, that identifies exactly where efforts to shorten the delay should be directed. We therefore carried out this systematic review and meta-analysis comparing the time from symptom onset to diagnosis in bulbar-onset versus limb-onset ALS. Because disease-modifying treatment begins only after diagnosis and survival in ALS is measured in months, quantifying this difference has direct clinical significance for efforts to reduce the delay to treatment. This review compiles and synthesizes the available evidence on diagnostic delay in bulbar-onset versus limb-onset ALS, pooling reported estimates into a single comparison and rates how reliable the overall estimate is.

## Review

Materials and methods

Protocol and Registration 

This systematic review and meta-analysis was conducted and reported in accordance with the Preferred Reporting Items for Systematic Reviews and Meta-Analyses (PRISMA) 2020 guidelines. The protocol was registered prospectively on the International Prospective Register of Systematic Reviews (PROSPERO) prior to the full search (Registration Number: CRD420261365399).

*Eligibility Criteria* 

Studies were eligible if they: (1) included adult patients (≥18 years) diagnosed with ALS using established criteria (El Escorial, Revised El Escorial, Awaji, or Gold Coast); (2) reported data separately for bulbar-onset and limb-onset (or spinal-onset) ALS; (3) reported time from symptom onset to confirmed diagnosis as a quantifiable outcome in months or years; (4) used a cohort, cross-sectional, registry-based, or randomized design; and (5) were published in English. Studies were excluded if they were case reports, series, reviews, editorials, letters, or abstracts without extractable data; did not distinguish onset types; did not report time to diagnosis; reported exclusively familial ALS; or reported only a segment of the diagnostic pathway. 

Search Strategy 

We searched two electronic databases, PubMed (MEDLINE) and the Cochrane Central Register of Controlled Trials (CENTRAL), from inception through April 2026, with no date restriction. The search combined three concept blocks using Boolean operators: (1) amyotrophic lateral sclerosis and its synonyms (e.g., motor neuron disease); (2) site of onset (bulbar, limb, spinal); and (3) diagnostic delay (time to diagnosis, diagnostic interval, and related terms). The full search strategy, applied across both databases, is provided in the Appendices.

To identify additional eligible studies, we screened the reference lists of all included articles (backward citation searching) and performed forward citation searching on key studies. The database search identified 351 records from PubMed and 1 from Cochrane CENTRAL, and a further three records were identified through citation searching.

Study Selection 

Records were imported into Rayyan (Rayyan Systems Inc., Cambridge, MA, USA), and screening was conducted both within Rayyan and manually using Microsoft Excel (Microsoft Corporation, Redmond, WA, USA). Titles and abstracts were independently screened by two reviewers, followed by full-text review. Any disagreements were resolved through discussion until consensus was reached. The PRISMA2020 flow diagram tool was used to document the selection process [6]. 

Data Extraction and Handling

Two reviewers independently extracted the first author, year, country, design, study period, diagnostic criteria, sample sizes, and the mean or median time from symptom onset to diagnosis for each onset group with the corresponding standard deviation (SD) or interquartile range (IQR). Each extracted value was verified against the source publication. 

Several studies did not report a single combined limb-onset group but instead reported upper-limb and lower-limb (or upper- and lower-extremity) onset separately [7-9]. For these, the two subgroups were combined into one limb-onset group using the standard formulae for pooling means and standard deviations, rather than by averaging the reported summary values. Where a study gave only a median and interquartile range (IQR), the corresponding mean and standard deviation (SD) were approximated using the Wan et al. [10] (mean ≈ (Q1 + median + Q3) / 3; SD ≈ (Q3 − Q1) / 1.35, the large-sample approximation). 

Four studies reported no usable measure of dispersion for either onset group: one reported means stratified by sex and onset without a measure of dispersion [11], two reported subgroup medians without a measure of spread [12,13], and one reported a median with full range only, from which a reliable variance could not be derived [14]. Because a within-study variance cannot be derived from these data, these studies could not be weighted in a meta-analysis and were summarized narratively. The Medicare-based study [13] was additionally limited by its reliance on administrative claims, in which the site of onset is not clinically validated. 

Risk of Bias Assessment 

Two reviewers independently evaluated the methodological quality of the included observational studies using the Newcastle-Ottawa Scale (NOS) [15], which assesses selection (up to 4 stars), comparability (up to 2 stars), and outcome (up to 3 stars), with a maximum possible score of nine stars. Comparability stars were awarded only where a study adjusted for or matched on key demographic confounders (notably age and sex) when comparing onset groups; studies reporting unadjusted descriptive comparisons did not receive a comparability star. Studies scoring 7-9 were classified as low risk, 4-6 as moderate, and below 4 as high. Disagreements were resolved by consensus. 

Statistical Analysis

Meta-analysis used a random-effects model, with between-study variance estimated by the DerSimonian and Laird method [16]. The outcome was the weighted mean difference (MD) in time from symptom onset to diagnosis (bulbar minus limb, in months); a negative MD indicates a shorter delay in bulbar onset. Results are reported with corresponding 95% confidence intervals (CIs). The analysis included the nine studies with a usable measure of dispersion by onset group; the remaining four studies were summarized narratively. Heterogeneity was quantified using the I² statistic and Cochran Q test (I² of 0-40% low, 41-60% moderate, above 60% substantial). Robustness was assessed by leave-one-out analysis. Formal funnel-plot asymmetry testing (e.g., Egger's) was omitted, since such tests lack power and reliability when fewer than 10 studies are pooled; publication bias could therefore not be formally assessed and cannot be excluded. All analyses were performed in Review Manager (RevMan), Version 5.4 (The Cochrane Collaboration, London, UK), using the inverse-variance random-effects method [17]. The certainty of evidence for the pooled comparison was rated using the Grading of Recommendations, Assessment, Development and Evaluations (GRADE) approach [18], considering risk of bias, inconsistency, indirectness, imprecision, and publication bias. Two reviewers independently applied GRADE, and disagreements were resolved by consensus. Because the review addressed a single continuous outcome from observational cohorts, certainty was summarized as a GRADE evidence profile rather than a full Summary of Findings table.

Results 

*Study Selection* 

**Figure 1:**
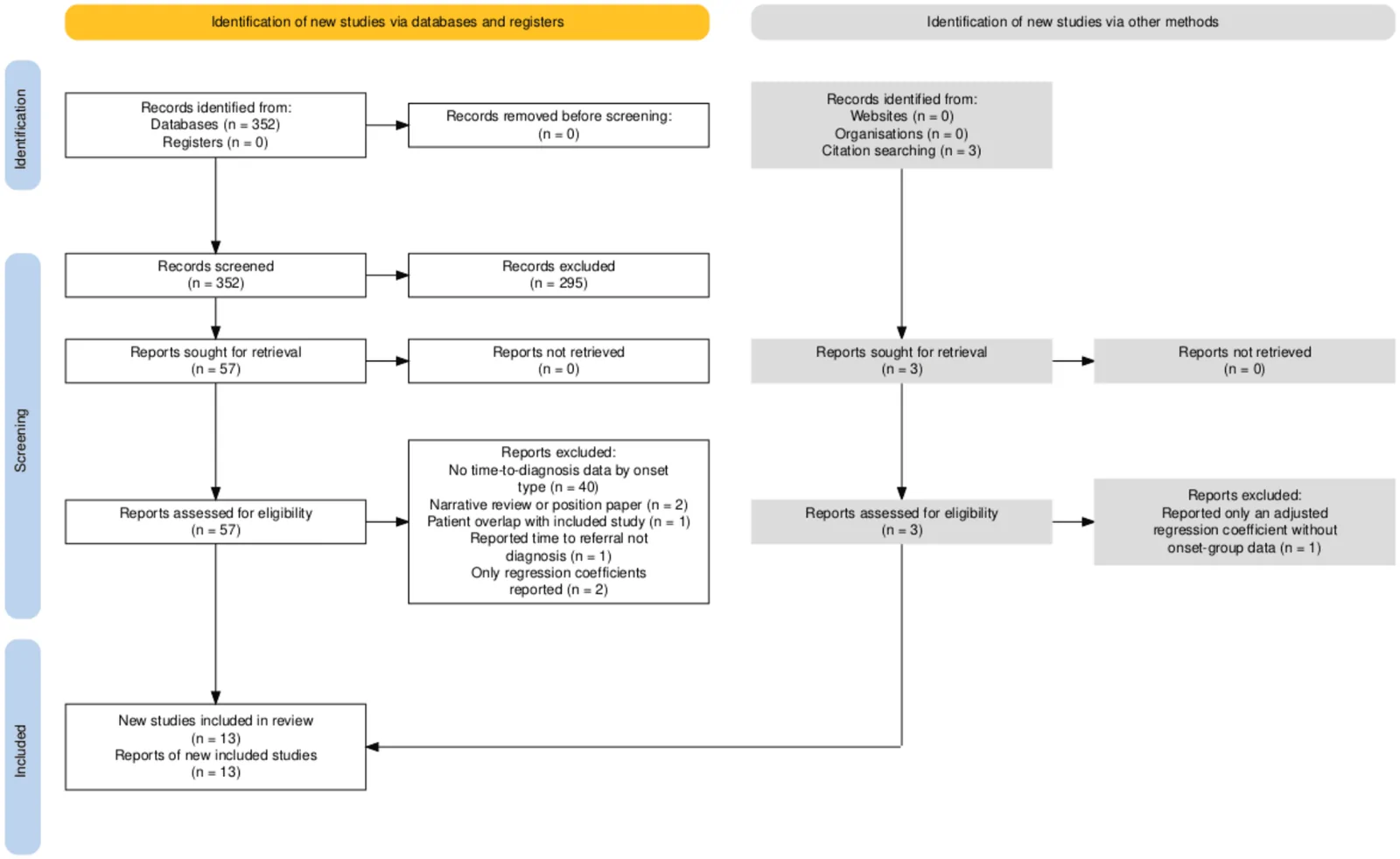
PRISMA 2020 flow diagram of study selection. PRISMA: Preferred Reporting Items for Systematic Reviews and Meta-Analyses

Database searches identified 352 records (PubMed, n = 351; Cochrane CENTRAL, n = 1). No duplicates were identified. Screening of titles and abstracts removed 295 records, leaving 57 reports for full-text assessment, of which 46 were excluded: 40 did not report time to diagnosis stratified by onset type, two were narrative reviews or position papers without original data [19,20], one had patient overlap with an included study (the same institution and study period as the included Valencian cohort [9]), one reported time to neurology referral rather than time to diagnosis, and two reported only regression coefficients without extractable subgroup data. The excluded full-text studies and their reasons for exclusion are detailed in Figure 1. Database searching yielded 11 studies. Citation searching (backward and forward) identified three further reports assessed at full text, of which two met the inclusion criteria, and one was excluded for reporting only an adjusted regression coefficient without onset-group data. A total of 13 studies were included in the review; nine contributed to the quantitative synthesis, and four were summarized narratively (Figure 1).

Study Characteristics 

The 13 included studies were published between 2000 and 2023 and encompassed data from eight countries: Japan [11,21], Italy [14,22,23], the USA [7,12,13], Sweden [8], Spain [9], the United Kingdom [24], Egypt [25], and Germany [26]. The total included sample comprised 898 bulbar-onset and 2,438 limb-onset patients. Three studies [7-9] reported upper- and lower-limb onset separately; these subgroups were combined as described above. Study characteristics are summarized in Table 1. The conversions and subgroup pooling applied are detailed in Table 2.

**Table 1:** Characteristics of included studies and extracted diagnostic-delay values. Characteristics of included studies and extracted diagnostic-delay values. Where upper- and lower-limb onset were reported separately, both are shown; they were combined into a single limb-onset group for analysis (Table 2). *Subgroup means with no subgroup SD; the overall cohort SD (11.2) was used as an approximation. No measure of dispersion was reported by onset group for the studies marked with a dagger (†); these were not pooled and were summarized narratively. ‡Zoccolella [14] reported a median with full range only; the range-based conversion produced an implausible reconstructed limb-onset mean (approximately 23 months versus a reported median of 10) owing to an extreme upper value, so this study was not pooled and was summarized narratively. mo = months; SD = standard deviation; IQR = interquartile range; UL/UE = upper limb/extremity; LL/LE = lower limb/extremity; Retro. = retrospective; Pop. = population; Assoc. = association; vets = veterans; neurol. = neurological; Rev. = Revised; WFN = World Federation of Neurology; EEC = El Escorial criteria; AHC = Airlie House criteria; ICD-9 = International Classification of Diseases, Ninth Revision.

Study	Country	Design	Bulbar, n	Bulbar delay (months, mo)	Limb, n	Limb delay (mo)	Measure	Criteria
Kano 2013 [21]	Japan	Retrospective (Retro.) cohort	78	9.2 (SD 4.5)	124	15.2 (SD 7.7)	Mean+SD	Revised El Escorial
Vázquez-Costa 2021 [9]	Spain	Retro. cohort	46	12.7 (SD 9.67)	94	upper limb (UL) 11.73 (8.35); lower limb (LL) 14.47 (8.99)	Mean+SD	El Escorial
Rashed 2020 [25]	Egypt	Clinic cohort	9	8.2 (SD 2.57)	21	22.95 (SD 17.6)	Mean+SD	Rev. El Escorial
Kraemer 2009 [26]	Germany	Retro. cohort	35	9.0 (SD 6.5)	65	16.4 (SD 17.5)	Mean+SD	Rev. El Escorial
Cellura 2012 [22]	Italy	Retro. cohort	65	10 (IQR 6-15)	195	12 (IQR 7-23)	Median+IQR	El Escorial/World Federation of Neurology (WFN)
Palese 2019 [23]	Italy	Population (Pop.)-based	47	10.3 (IQR 7.1-14.9)	87	12.4 (IQR 7.1-22)	Median+IQR	Clinical (neurol.)
Sennfält 2023 [8]	Sweden	Pop.-based	136	11.7 (IQR 7.4-16)	202	upper extremity (UE) 12.9 (8.8-17.8); lower extremity (LE) 16.0 (9.4-27.5)	Median+IQR	El Escorial
Khishchenko 2010 [7]	USA	Registry (vets)	258	9 (IQR 5-14)	1056	UE 10 (6-20); LE 13 (7-25)	Median+IQR	Rev. El Escorial
Househam 2000 [24]	UK	Association (Assoc.) cohort	14	11.6	37	16.6 (UL+LL)	Mean*	Clinical (neurol.)
Paganoni 2014 [12]	USA	Retro. cohort	84	9	196	12	Median†	International Classification of Diseases, Ninth Revision (ICD-9) chart
Williams 2013 [13]	USA	Claims (≥65)	46	15	201	30	Median†	ICD-9 (Medicare)
Iwasaki 2002 [11]	Japan	Retro. cohort	55	10.1	62	14.0	Mean†	WFN criteria
Zoccolella 2006 [14]	Italy	Pop.-based	25	7.0 (range 3.8-49.2)‡	98	10.0 (range 1-70.7)‡	Median+range	El Escorial criteria (EEC)/Airlie House criteria (AHC)
Total			898		2438			

**Table 2:** Conversions and subgroup pooling used to derive analyzed means and SDs. Conversions and subgroup pooling used to derive analyzed means and SDs. Median (IQR) values were converted to mean and SD using the method of Wan et al. [10]: mean ≈ (Q1 + median + Q3)/3; SD ≈ (Q3 − Q1)/1.35. Vázquez-Costa [9] reported subgroup means (SD) directly and required no conversion. Where upper- and lower-limb (or upper- and lower-extremity) subgroups were reported separately, they were combined into a single limb-onset group using the standard pooled-mean and pooled-SD formulae, weighted by subgroup sample size (n). Both onset groups are shown for every study requiring a conversion; studies reporting mean (SD) directly by onset group (Kano [21], Rashed [25], Vázquez-Costa bulbar [9], Househam [24]) are given in Table 1. Paganoni 2014 [12] and Williams 2013 [13] reported no measure of dispersion by onset group and could not be pooled. UL/UE = upper limb/extremity; LL/LE = lower limb/extremity; mo = months

Study	Onset group	Reported value(s)	Derived mean (mo)	Derived SD (mo)
Cellura 2012 [22]	Bulbar	Median 10 (IQR 6–15)	10.33	6.67
Cellura 2012 [22]	Limb	Median 12 (IQR 7–23)	14.00	11.85
Palese 2019 [23]	Bulbar	Median 10.3 (IQR 7.1–14.9)	10.77	5.78
Palese 2019 [23]	Limb	Median 12.4 (IQR 7.1–22)	13.83	11.04
Vázquez-Costa 2021 [9]	Limb (UL+LL)	UL (n=37): mean 11.73 (SD 8.35); LL (n=57): mean 14.47 (SD 8.99)	13.39	8.80
Sennfält 2023 [8]	Bulbar	Median 11.7 (IQR 7.4–16)	11.70	6.37
Sennfält 2023 [8]	Limb (UE+LE)	UE (n=85): median 12.9 (IQR 8.8–17.8); LE (n=117): median 16.0 (IQR 9.4–27.5)	15.75	11.28
Khishchenko 2010 [7]	Bulbar	Median 9 (IQR 5–14)	9.33	6.67
Khishchenko 2010 [7]	Limb (UE+LE)	UE (n=539): median 10 (IQR 6–20); LE (n=517): median 13 (IQR 7–25)	13.47	12.00

Risk of Bias

Newcastle-Ottawa Scale scores ranged from 5 to 8 stars (Table 3).

**Table 3:** Newcastle-Ottawa Scale risk-of-bias assessment. Newcastle-Ottawa Scale (NOS) risk-of-bias assessment. Selection: S1 representativeness; S2 selection of non-exposed; S3 ascertainment of exposure; S4 outcome not present at start. Comparability: C1 (age/sex); C2 (additional factor). Outcome: O1 assessment; O2 follow-up duration; O3 follow-up completeness. A filled cell (1) = one NOS star awarded. Comparability stars were awarded only where the study adjusted for or matched on key confounders when comparing onset groups; studies reporting unadjusted descriptive comparisons did not receive one. The NOS does not capture the population-specific selection bias of the veterans registry (Khishchenko 2010 [7]) and the Medicare cohort (Williams 2013 [13], restricted to patients aged >=65 years). Studies scoring 7-9 were classified as low risk, 4-6 as moderate, and below 4 as high.

Study	S1	S2	S3	S4	C1	C2	O1	O2	O3	Total
Kano 2013 [21]	1	1	1	1			1	1		6
Vázquez-Costa 2021 [9]	1	1	1	1	1		1	1	1	8
Rashed 2020 [25]		1	1	1			1	1		5
Kraemer 2009 [26]	1	1	1	1			1	1		6
Cellura 2012 [22]	1	1	1	1			1	1		6
Palese 2019 [23]	1	1	1	1	1	1	1	1		8
Sennfält 2023 [8]	1	1	1	1	1	1	1	1		8
Khishchenko 2010 [7]		1	1	1	1		1	1	1	7
Paganoni 2014 [12]	1	1	1	1	1		1	1		7
Househam 2000 [24]		1	1	1			1	1		5
Williams 2013 [13]		1		1			1	1	1	5
Iwasaki 2002 [11]	1	1	1	1			1	1		6
Zoccolella 2006 [14]	1	1	1	1			1	1		6

Five studies were rated low risk of bias (7-9 stars) and eight moderate risk (4-6 stars); none were high risk. Comparability stars were awarded only where a study formally adjusted, matched, or stratified the bulbar-versus-limb delay comparison for age or sex; studies reporting unadjusted descriptive comparisons did not receive one. The veterans-registry study [7] and the Medicare study [13] carried population-specific selection biases, a predominantly male veterans population and a cohort restricted to patients aged ≥65 years, that are not fully captured by the NOS; in the Medicare study [13], onset type was additionally derived from administrative claim codes rather than clinical assessment. 

Meta-Analysis

**Figure 2:**
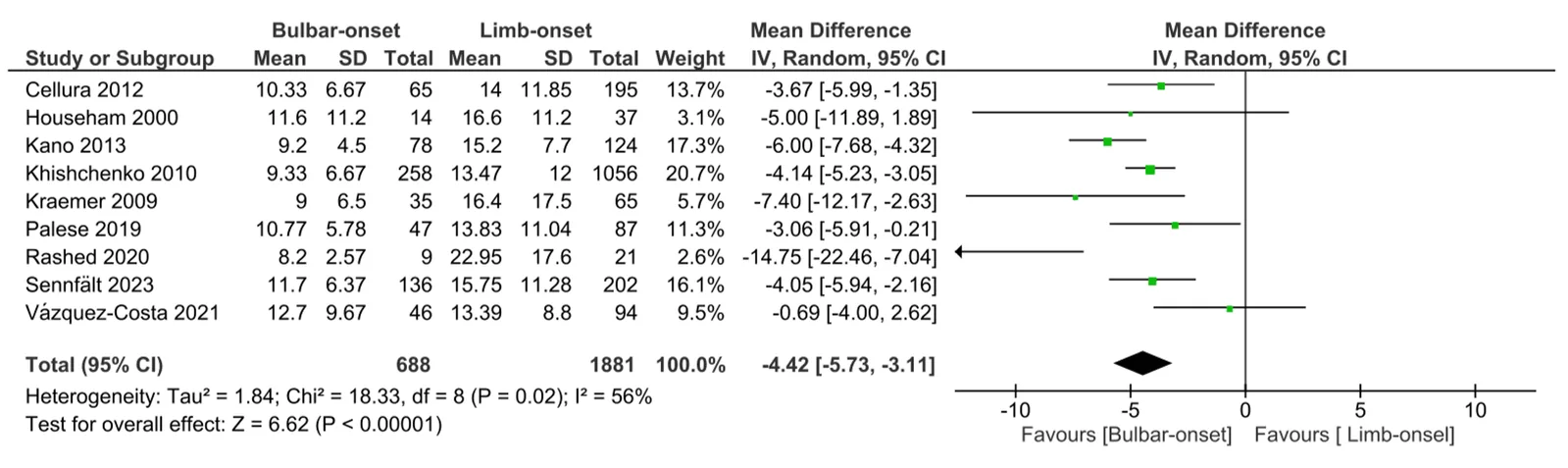
Forest plot of the mean difference in time from symptom onset to diagnosis, bulbar-onset versus limb-onset amyotrophic lateral sclerosis (ALS) Random-effects inverse-variance model; nine pooled studies; generated in RevMan 5.4 (The Cochrane Collaboration, London, UK). A negative mean difference indicates faster diagnosis in bulbar-onset disease. Cellura [22], Househam [24], Kano [21], Khishchenko [7], Kraemer [26], Palese [23], Rashed [25], Sennfält [8], Vázquez-Costa [9].

All nine pooled studies reported a shorter diagnostic delay in bulbar-onset than in limb-onset patients. The random-effects estimate showed that bulbar-onset patients were diagnosed significantly faster (MD = −4.42 months; 95% CI −5.73 to −3.11; Z = 6.62; p < 0.001). Heterogeneity was moderate (I² = 56%; tau-squared (τ²) = 1.84; Q = 18.33; degrees of freedom (df) = 8; p = 0.019). Individual study estimates ranged from −0.69 months (95% CI −4.00 to 2.62) [9] to −14.75 months (95% CI −22.46 to −7.04) [25]. The forest plot is presented in Figure 2.

Robustness 

On leave-one-out analysis, the pooled estimate remained stable, ranging from −4.08 months (omitting Kano [21]) to −4.74 months (omitting Vázquez-Costa [9]), and remained statistically significant in every iteration, indicating that no single study drove the result. 

Studies Not Included in the Pooled Analysis

Four studies meeting the inclusion criteria could not be entered into the meta-analysis because they reported no usable measure of dispersion by onset group. All four were directionally consistent with the pooled result. One study [11] reported a mean diagnostic interval of 10.1 months in bulbar-onset versus 14.0 months in limb-onset patients (pooled across sex); another [12] reported median total diagnostic times of 9 versus 12 months, respectively; and a Medicare-based study [13] reported median times of 15 versus 30 months. This last study [13] is further limited by its reliance on administrative Medicare claims restricted to patients aged ≥65 years, in which the site of onset is not clinically validated; its estimate should therefore be interpreted with particular caution. A population-based Italian registry series [14] reported a shorter median diagnostic interval in bulbar-onset (7 months; range 3.8-49.2) than in limb-onset patients (10 months; range 1-70.7); because only a median with full range was available and the range-based conversion produced an implausibly high reconstructed limb-onset mean, this study was summarized narratively rather than pooled. The consistent direction of these four studies supports, but does not add statistical weight to, the pooled finding.

*Certainty of Evidence (GRADE)* 

The certainty of evidence for the difference in diagnostic delay between bulbar-onset and limb-onset ALS was rated as very low using the GRADE approach [18] (Table 4).

**Table 4:** GRADE summary of findings. GRADE summary of findings: certainty of evidence for the difference in diagnostic delay, bulbar-onset versus limb-onset amyotrophic lateral sclerosis (ALS). Certainty for a body of observational evidence begins at "low" and may be rated down for serious concerns or up for special strengths (e.g., a large effect). One domain was rated down (risk of bias); heterogeneity, although moderate, was consistent in direction and considered explainable, and was not rated down. The resulting overall rating is very low certainty: the true difference is likely in the observed direction, but its precise magnitude is uncertain. GRADE = Grading of Recommendations, Assessment, Development and Evaluations.

Domain	Assessment
Outcome	Time from symptom onset to diagnosis (months), bulbar vs limb onset
Studies/participants	9 observational studies pooled (4 further studies summarized narratively); 2,569 patients (688 bulbar, 1,881 limb)
Pooled effect	Mean difference −4.42 months (95% CI −5.73 to −3.11), favouring earlier diagnosis in bulbar onset
Starting certainty	Low (body of observational evidence)
Risk of bias	Serious - predominantly retrospective designs reliant on recalled onset dates (rated down one level)
Inconsistency	Not serious - moderate statistical heterogeneity, I² = 56%
Indirectness	Not serious - studies directly address the review question
Imprecision	Not serious - narrow pooled confidence interval excluding the null
Publication bias	Not assessed — fewer than 10 pooled studies precluded formal testing
Large-effect upgrade	Not applied (conservative)
Overall certainty	VERY LOW

As a body of observational evidence, certainty began at a low level. It was not downgraded for indirectness, as the included studies addressed the review question directly, nor for imprecision, as the pooled confidence interval was narrow and excluded the null. Certainty was rated down one level for risk of bias, as the included studies were predominantly retrospective and reliant on recalled symptom-onset dates. Heterogeneity, although moderate (I² = 56%), was consistent in direction across all studies and was considered explainable by differences in era, healthcare system, and diagnostic criteria; certainty was therefore not additionally downgraded for inconsistency. Publication bias could not be formally assessed because fewer than 10 studies were pooled. Although the effect was consistent in direction across all studies, no upgrade for a large effect was applied. The overall rating of very low certainty indicates that the true difference is likely in the observed direction, while its precise magnitude remains uncertain.

Discussion

This systematic review and meta-analysis shows that bulbar-onset ALS is diagnosed about four months earlier than limb-onset ALS (pooled mean difference −4.42 months; 95% CI −5.73 to −3.11). Every included study pointed in the same direction; the pooled result stayed stable on leave-one-out testing, and the four studies that could not be pooled agreed. Given the median survival of only 2-4 years after symptom onset [1,3], a four-month diagnostic delay is clinically meaningful because it postpones disease-modifying treatment and multidisciplinary care. To our knowledge, this is the first review to directly compare time to diagnosis between the two forms; earlier studies reported them separately but never pooled them, so the size of the gap had not been measured before.

The gap is best understood within the wider problem of diagnostic delay in ALS, where the total interval is usually about 9 to 18 months [3] because there is no confirmatory test, diagnosis rests on clinical criteria, and early symptoms mimic commoner conditions [19,20]. That interval splits into patient help-seeking time and health-system time to referral and diagnosis, and our findings suggest the difference between the forms lies mostly in the system part: bulbar symptoms route patients quickly to ENT or neurology, whereas limb weakness is common and vague and is often sent down non-neurological paths first. Several included studies support this, showing that limb-onset patients pass through orthopedic, spine, and rheumatology services before reaching a neurologist [8,9,21].

The limb-onset misattribution pathway deserves emphasis, because this is where the extra delay can be recovered. Focal, painless, slowly worsening weakness is easily blamed on the common compressive problems clinicians see every day: hand or finger onset is attributed to cervical radiculopathy, cervical spondylosis, carpal tunnel syndrome, or ulnar neuropathy, and leg onset to lumbar radiculopathy or peripheral neuropathy [1]. Patients may undergo months of physiotherapy, repeated scans, and sometimes surgery before ALS is recognized; Kano and colleagues found that limb-onset patients seen first by an orthopedist waited longer for a diagnosis [21]. Certain features should prompt neurological referral: weakness that keeps progressing rather than staying stable, wasting and twitching spreading beyond a single nerve or root, mixed upper and lower motor neuron signs, no sensory loss or pain, and no improvement after surgery or imaging that does not match how sick the patient is [1,3].

Bulbar-onset ALS is diagnosed faster not because it is easy to recognize: slurred speech and swallowing problems are themselves mistaken for stroke, myasthenia gravis, or ENT disease [1,3], but because these symptoms are unusual, distressing, and obvious, alarming patients, families, and doctors enough to speed up referral. The contrast between the two forms is therefore less about diagnostic difficulty than about which symptom raises alarm and which specialty the patient is sent to.

These findings are directly actionable. Because most of the excess delay in limb-onset ALS builds up before the patient reaches a neurologist, the highest-yield targets are the first-contact clinicians, primary care physicians, orthopedic and spine surgeons, rheumatologists, and physiotherapists. Simple referral rules for progressive, one-sided, painless limb weakness without a clear structural cause, together with rechecking a structural diagnosis when the patient does not improve as expected, could shorten the delay. The payoff is concrete: earlier diagnosis widens the window to start riluzole, edaravone, and, in *SOD1*-related disease, tofersen [3,5], to enter a multidisciplinary clinic, and to plan ahead.

A long, winding path to diagnosis also carries an emotional toll that delay figures miss. Months of inconclusive tests, wrong diagnoses, and treatments that do not help cause worry and uncertainty and can wear down trust in doctors, while the eventual correct diagnosis often brings both relief at being believed and the shock of the diagnosis itself, now with less time left. For families, the delay diverts energy into the search for answers instead of adjustment and shortens the time the patient can still speak, move, and share in their own decisions. It also compresses time-sensitive planning, work and finances, home changes, care arrangements, feeding tubes, breathing support, and advance directives, which is far easier done calmly than in a crisis. Seeing misdiagnosis as lost planning and coping time, not only lost treatment time, makes reducing the limb-onset delay a way to improve quality of life for patients and families, not just survival [3].

Two further points deserve note. First, the gap should be read against a changing background: the included studies span more than twenty years and several versions of the diagnostic criteria, and the newer Gold Coast criteria, by requiring less proof of spread, may shorten delay and could narrow the gap; whether this has happened is unknown and could be answered by future studies that report results by onset type. Second, the moderate variation between studies (I² = 56%) reflects differences in era, health system, and criteria rather than any disagreement in direction, and factors such as sex, country, and access to neurology may also modify delay and deserve dedicated study.

Taken together, these results should be read with some caution about the exact figure, yet they add what the individual studies could not. Until now, each cohort reported its own numbers, and the sense that bulbar-onset ALS is diagnosed sooner rested on scattered, single-setting findings that had never been compared directly. Pooling them shows the pattern is not a quirk of one health system or one era: it holds across all 13 studies, eight countries, and more than twenty years; the combined estimate rules out "no difference," and it stays stable when any single study is removed. Only the exact size of the gap remains uncertain - about four months here, though the very low GRADE rating means the true value may be somewhat higher or lower, mainly because the source studies are retrospective and rely on remembered onset dates. The value of this meta-analysis is therefore to turn a widely held clinical impression into a single, tested estimate, and to show that the delay is large enough, measured in months, to justify targeted awareness efforts for limb-onset ALS and routine reporting of diagnostic delay by onset type.

Limitations 

Several limitations should be acknowledged. First, most included studies were retrospective and relied on patient or clinician recall of symptom onset, which may introduce recall bias, particularly for slowly progressive limb-onset cases. Second, four studies reported medians with IQRs rather than means and SDs; for these, we applied the method of Wan et al. [10], which assumes approximately normal data, whereas diagnostic-delay distributions are typically right-skewed; the reconstructed means may therefore be biased, and the SDs may not fully capture dispersion. Third, four studies [11-14] reported no measure of dispersion by onset group; they were summarized narratively and were each directionally consistent with the pooled estimate. Fourth, several studies did not report a single limb-onset group, requiring upper- and lower-limb subgroups to be pooled; although done with standard formulae, this introduces a derived rather than directly reported estimate. Fifth, the review was restricted to English-language publications, and only PubMed and Cochrane CENTRAL were searched owing to limited access to Embase, Scopus, and Web of Science, so some studies may have been missed. Sixth, because fewer than 10 studies were pooled, formal tests for funnel-plot asymmetry could not reliably be applied, and publication or small-study bias cannot be excluded. Finally, the definition of limb onset varied across studies, with some combining spinal onset into the limb category. 

Future directions 

Future research should examine whether the diagnostic-delay differential between onset types has narrowed over time as ALS awareness has improved, and whether newer criteria such as the Gold Coast criteria have affected time to diagnosis. Prospective, registry-based studies with standardized delay measurement and consistent reporting of dispersion by onset group would substantially strengthen future syntheses. Examining diagnostic delay by sex and geographic region as potential effect modifiers may also identify subpopulations at greatest risk.

## Conclusions

This systematic review and meta-analysis demonstrates that bulbar-onset ALS is diagnosed approximately 4 months earlier than limb-onset ALS, a finding consistent across studies from eight countries and robust to leave-one-out analysis. These findings derive predominantly from retrospective observational studies and should be interpreted alongside the very low GRADE certainty rating, which reflects uncertainty in the precise magnitude of the delay rather than its direction. Given that total survival in ALS is measured in months, this differential is clinically significant. The results support targeted awareness strategies directed at the clinicians most likely to encounter limb-onset presentations, including primary care physicians, orthopedic surgeons, and spine specialists, to reduce diagnostic delay and maximize the therapeutic window for available treatments.

## Appendices

Electronic search strategies

Databases searched: PubMed, Cochrane Library

Search date: 11 April 2026

Coverage: Database inception to 11 April 2026

Results: PubMed - 351 results, Cochrane Library - 1 result 

Search String :

("amyotrophic lateral sclerosis" OR "ALS" OR "motor neuron disease" OR "motor neurone disease" OR "MND") AND ("bulbar onset" OR "bulbar-onset" OR "bulbar onset ALS" OR "bulbar phenotype" OR "bulbar presentation" OR "limb onset" OR "limb-onset" OR "limb onset ALS" OR "spinal onset" OR "site of onset" OR "onset phenotype" OR "onset type") AND ("diagnostic delay" OR "time to diagnosis" OR "time till diagnosis" OR "time until diagnosis" OR "delay in diagnosis" OR "diagnosis delay" OR "diagnostic interval" OR "time from symptom onset to diagnosis" OR "symptom onset to diagnosis" OR "pre-diagnostic delay" OR "diagnostic latency" OR "delay to diagnosis" OR "time from onset to diagnosis")
